# Inflammatory Bowel Disease: A Personalized Approach

**DOI:** 10.3389/fped.2020.620545

**Published:** 2021-02-11

**Authors:** Anastasia Konidari, David Dickens, Munir Pirmohamed

**Affiliations:** ^1^B Pediatric Clinic, Paidon Aglaia Kyriakou Children's Hospital, Athens, Greece; ^2^The Wolfson Centre for Personalized Medicine, Institute of Systems, Molecular and Integrative Biology (ISMIB), University of Liverpool, Liverpool, United Kingdom

**Keywords:** inflammatory bowel disease, personalized medicine, therapeutics, genetic susceptibility, childhood

## Introduction

Inflammatory bowel disease (IBD) encompasses three major forms of chronic intestinal inflammation, including Crohn's disease (CD), ulcerative colitis (UC), and unclassified IBD (IBDU), previously known as indeterminate colitis. The definition of clinical phenotypes follows internationally agreed standards based on a constellation of clinical, endoscopic, radiologic, and histologic features. IBD is recognized as a heterogeneous spectrum of disorders with distinct clinical courses and symptom chronicity, which may present anytime from early childhood to adulthood (1–3). The burden of disease can be modified by treatment, but in principle, IBD presents with relapsing and remitting course throughout life (4). CD and UC, while sharing several similar pathologic and clinical features, have distinct differences in prognosis and management (5). Disease type and distribution in part influence disease progression and prognosis (6, 7).

Pediatric IBD is associated with greater disease burden and morbidity because it affects children at a critical period for growth and development. It is characterized by extensive and severe disease, which, if untreated, results in increased risk of intestinal strictures necessitating surgical treatment (8), growth failure, nutritional deficiencies, delayed puberty, metabolic bone disease, and increased risk of extra-intestinal manifestations such as hepatic, eye and skin disease, and psychiatric morbidity (9–11).

## Genetic Susceptibility

IBD is a heterogeneous disorder with multifactorial etiology (12); mapping of the interaction between genetic and environmental factors may inform molecular classification of disease in the future (13, 14). Over the last decade, various studies have reported that multiple genomic loci increase genetic susceptibility to IBD (15). Genome-wide association (GWA) and other studies have demonstrated how genetics can be applied for phenotype stratification (16, 17); such studies have provided an overview of relative contribution of various genomic loci to a number of human diseases including IBD, through genotyping of a large number of single-nucleotide polymorphisms (SNPs) across the human genome (18).

More than 163 IBD susceptibility genetic loci have been found in cohorts of European descent (19), with emerging data of how genetic variation can determine disease distribution (20). Of these loci, 110 confer risk to both IBD subtypes, whereas 30 and 23 loci are unique to CD and UC, respectively. The 163 loci explain 13.6% of CD and 7.5% of UC total disease variance, respectively (19). Liu et al. (21) have conducted the first trans-ethnic association study in IBD with use of GWA. Immunochip data from 86,640 Europeans and 9,846 subjects of Asian descent revealed additional 38 IBD susceptibility loci; the majority show similar direction and magnitude of effect between European and non-European populations (21).

Susceptibility genes encode proteins such as the nucleotide-binding oligomerization domain protein 2 (NOD2) with mutations affecting nuclear factor-kappa beta (NFKB) production, autophagy-related 16-like protein 1 (ATG16L1), immunity-related GTPase family M (IRGM), functional toll-like receptors (TLRs) (22), signal transducer and activation of transcription 3 (STAT3) protein, and interleukin 23 receptor (IL-23R) (23). Epigenetic alterations, such as DNA methylation, may further influence disease progression (24).

## Childhood-Onset Inflammatory Bowel Disease

IBD incidence and prevalence in children are increasing (25). National cohort studies have been included in a recent systematic review, which has confirmed the rising global trend of pediatric IBD in both developed and developing countries (26). Emerging evidence supports the need for a “top-down” approach in the implementation of treatment modalities, especially in children (27, 28). Furthermore, population-based cohort studies showed twice the risk of developing corticosteroid (CS) dependency within the first year from diagnosis in children, when compared with adults (29). Early poor response to treatment and severe disease extent at diagnosis as predictors of colectomy in pediatric UC further support the need for early initiation of patient-tailored treatment.

Until fairly recently, there has been a paucity of studies about genes predisposing to childhood-onset IBD. A pediatric GWA study by Cutler et al. (30) in 1,008 pediatric-onset IBD patients and 1,633 controls confirmed overlap with common adult IBD susceptibility loci such as NOD2 and IL23R. Denson et al. (31) reported the results of whole-exome sequencing (WES) in 543 children with IBD. Low/normal neutrophil intrinsic granulocyte macrophage colony-stimulating factor (GM-CSF) signaling was found to be associated with colony-stimulating factor 2 receptor A subunit (CSF2RA) missense mutations, where neutrophils from IBD patients exhibited alterations in gene expression regulating cytokine production, wound healing, cell survival, and proliferation (31). The same group performed WES in children with IBD and healthy controls to identify mutations in genes encoding nicotinamide adenine dinucleotide phosphate oxidase (NADPH), and neutrophil gene expression associated with reactive oxygen species production. Patients with specific mutations in NADPH oxidases had more aggressive disease course in CD (31).

Very-early-onset IBD (VEO-IBD) presents below 6 years of age and manifests with a distinct phenotype, however, it is not predominantly a monogenic condition; next-generation sequencing such as WES or whole-genome screening (WGS) may help establish the molecular diagnosis to some extent, with a reported diagnostic yield varying between 13 and 26.5% (32), allowing for patient-specific early intervention the opportunity to screen family members for carrier detection and genetic counseling. IBD-like monogenic disorders are related to primary immunodeficiency, for example, defects of T and B lymphocyte selection and activation, disorders affecting regulatory T cell activity, and interleukin 10 (IL10) and IL10 receptor A/B (IL10R) A/B signaling (33). Other monogenic conditions include dysfunction of NADPH oxidase complex, X-linked inhibitor of apoptosis (XIAP), lipopolysaccharide responsive beige-like anchor protein (LRBA), cytotoxic T lymphocyte-associated protein 4 (CTLA-4), STAT3, and chronic granulomatous disease (34). Mutations in the epithelial cell adhesion molecule (EPCAM), myosin Vb (MYO5B), Fork head Box P3 gene (FOXBP3), Tetratricopeptide Repeat Domain 37 (TTC37) (35), and the “immune dysregulation, poly-endocrinopathy, enteropathy, X-linked” syndrome (36) have also been implicated in VEO-IBD. Tumor necrosis factor alpha-induced protein 3 (TNFAIP3) mutations cause infantile-onset IBD (37). Monogenic IBD is also caused by genes related to intestinal epithelial defects, such as intestinal epithelial cell adhesion and generation of reactive oxygen species (38).

## Modern Treatment Paradigms

Therapeutic approaches involve potent inhibition of inflammatory pathways and immune suppression. Complicated disease course is largely associated with choice of treatment agents, drug efficacy, and sustained treatment response or lack of it. Medications are therefore not universally effective and can be associated with significant toxicity (39).

Individual carriers of risk allele HLA DRB1 03:01 for instance have been shown to be at 3-fold risk of 5ASA-related nephrotoxicity (40).

Next-generation sequencing performed in leucocytes of children with IBD at diagnosis and 4 weeks following introduction of CS showed significantly altered expression of 18 micro-RNAs; three of these micro-RNAs contained glucocorticoid-responsive elements in their gene promoters and could be putatively directly regulated by the GC receptor, while others recognized 3′ untranslated region of the GR gene (41).

Thiopurine drug monitoring is a successful paradigm of personalized treatment. Thiopurine methyltransferase (TPMT) genotyping is undertaken routinely prior to thiopurine introduction in patients with IBD (42). Patient response to treatment exhibits inter-individual variability thought to result from variation in drug metabolism (43, 44). TPMT genotype and activity can partially explain the variability in patient response. Hematological toxicity, commonly manifesting as leukopenia, is associated with the homozygous recessive TPMT genotype and intermediate/low TPMT activity. Optimal 6TGN level is a surrogate marker of response to thiopurine treatment; low metabolite concentration is indicative of poor compliance (45). 6MMPR monitoring can be useful for early identification of liver toxicity (43).

NUDT15 exon mutations have been associated with thiopurine-induced leucopenia in Asian patients (46). Sutiman et al. (47) have reported higher risk of thiopurine-induced myelotoxicity in Asian patients with IBD. The Clinical Pharmacogenetics Implementation Consortium has published guidance on thiopurine dosing recommendations based on TPMT and NUDT15 genotypes (48). Thiopurine-induced pancreatitis has been related to HLA DQA1–HLA DRB1 homozygous haplotypes; however, this association has not been yet included in routine pre-treatment pharmacogenetic panels (49).

There is emerging evidence about benefits of early anti-TNF alpha administration (“top-down approach”) for early disease remission, avoidance of later progression to stricturing/penetrating disease in CD, improved quality of life, and reduction of hospital admission rates (50, 51). Primary loss of response to anti-TNF alpha occurs in up to a third of patients, with up to half of the patients losing response over time (52). There is preliminary evidence of HLA DQA1^*^05 haplotype association with 2-fold increase of anti-TNF alpha immunogenicity risk (53). The association of drug levels and antibodies in relation to clinical outcomes requires further investigation; however, recent systematic review and meta-analysis overall support the finding that regular therapeutic drug monitoring contributes to avoidance of higher disease relapse rate (54).

Consideration of variable efflux transporter expression associated with common genetic polymorphisms may also influence response to treatment (55). Clinical response to CS treatment for instance could be influenced by ABCB1 inter-individual expression variability on the basis that CSs are ABCB1 substrates (56).

## Discussion

IBD patients cannot be treated with “one size fits all” approach, because poor treatment response, adverse drug reactions, and therapeutic failure result in increasing morbidity and health-care costs (57). Loss of response or severe refractory disease remains the main challenges that clinicians and patients face during the disease course. Safety profile concerns such as organ toxicity, accumulated cancer risk, and drug interactions also influence clinician and patient choices. Best clinical outcome with fewer drug-related adverse reactions result in improved patient outcomes and optimal resource utilization; these goals are at the heart of personalized medicine. Research in patient-centered therapies based on the understanding of disease pathogenesis, genetic susceptibility, and inter-individual variability to treatment is ongoing (58, 59). The importance of personalized treatment in children is indicated by recent studies in childhood-onset CD, which have shown that non-sustained response to treatment may be more important in predicting disease relapse and overall complications than actual disease severity and extent at diagnosis (60).

Research that involves large international consortia of rigorously characterized patients is therefore paramount in order to identify and validate clinically meaningful and applicable biomarkers. This research requires large patient numbers of different ethnicities for identification of common and rare allele variants, with emphasis on genotype/phenotype characterization and monitoring of disease progression and treatment response. Cost issues pose further challenges in this endeavor. The next frontier in IBD therapeutics is about implementation of translational pharmacogenetic associations in treatment algorithms, in order to tailor pharmacotherapy to individual patient profiles (61).

## Author Contributions

AK: conceptualization, interpretation of data, and writing of original draft. DD and MP: manuscript reviewing and editing. All authors contributed to the article and approved the submitted version.

## Conflict of Interest

The authors declare that the research was conducted in the absence of any commercial or financial relationships that could be construed as a potential conflict of interest.
